# The role of dynamic enzyme assemblies and substrate channelling in metabolic regulation

**DOI:** 10.1038/s41467-018-04543-8

**Published:** 2018-05-30

**Authors:** Lee J. Sweetlove, Alisdair R. Fernie

**Affiliations:** 1 Department of Plant Sciences, University of Oxford, South Parks Road, Oxford, OX1 3RB UK; 2 Max-Planck-Institut für Molekulare Pflanzenphysiologie, Potsdam-Golm, 14476 Germany

## Abstract

Transient physical association between enzymes appears to be a cardinal feature of metabolic systems, yet the purpose of this metabolic organisation remains enigmatic. It is generally assumed that substrate channelling occurs in these complexes. However, there is a lack of information concerning the mechanisms and extent of substrate channelling and confusion regarding the consequences of substrate channelling. In this review, we outline recent advances in the structural characterisation of enzyme assemblies and integrate this with new insights from reaction–diffusion modelling and synthetic biology to clarify the mechanistic and functional significance of the phenomenon.

## Abstract (web summary)

Temporary association of metabolic enzymes is generally assumed to facilitate substrate channelling within the complex. In this review, Lee Sweetlove and Alisdair Fernie outline the nature and functional consequence of organising enzymes into assemblies, and discuss applications within the natural world and synthetic biology.

## Introduction

Metabolism is central to cellular function, growth and survival. Hundreds of chemical reactions occur simultaneously to process energy sources and to provide precursor molecules for cell construction and chemical defence. The majority of these reactions are accelerated and regulated by enzymes that not only allow metabolism to occur on an appropriate timescale for life but also allow the outputs of the metabolic system to be specified and controlled. Moreover, enzymes allow feedback loops and other regulatory mechanisms to operate so that the system can maintain homeostasis under variable conditions. Enzymes are highly effective catalysts and biochemical reactions are massively accelerated. Nevertheless, few enzymes have attained catalytic perfection and catalysis remains orders of magnitude slower than the diffusion of the substrates and products. Hence, the long-standing view of metabolic systems is that they are ‘well mixed’ with homogenously distributed enzymes and freely diffusing metabolites.

This view of metabolism is the main basis for modelling of enzyme kinetics^1^ and indeed holds true for purified enzymes in the test tube. However, over the past 40 years, evidence has steadily accumulated to suggest that this view is not an entirely accurate description of metabolism in vivo. Repeated observations have been made which suggest that enzymes are not always homogenously distributed in the cell but rather can come together in time and space, due to protein–protein interactions between them. The enzymes in such assemblies usually constitute recognised metabolic pathways and their formation and disassembly is dynamically responsive to biochemical conditions and stimuli. Moreover, metabolites may be channelled between sequential enzymes: i.e. the product of one enzyme is transferred to the next enzyme in the pathway without equilibrating with the bulk aqueous phase of the cell. In this case, the assemblies are known as metabolons, a term coined by Paul Srere some 40 years ago^2^ who provided the first detailed study of the phenomenon while working on the mitochondrial tricarboxylic acid (TCA) cycle. Since these pioneering studies, assemblies of consecutive enzymes have been observed in a wide variety of pathways, including glycolysis, oxidative phosphorylation, fatty acid, amino acid, polyketide, polyamine and polypeptide biosynthesis^3,4^. Enzyme assemblies have also been reported in photosynthesis and natural product metabolons in plants^5,6^ (see also Table 1 for details). Moreover, broad-scale screens using human cells have suggested the presence of up to 130,000 binary protein–protein interactions at any one time^7,8^, underlining the commonality of such interactions. It is now apparent that enzyme assemblies are a cardinal feature of metabolism across all domains of life and occur both in central metabolism and in the specialised metabolism of plants and fungi^6,9^ (Table 1).

**Table 1:** A representative list of dynamic enzyme assemblies, the species in which, and the method(s) by which, they were detected

Pathway	Method of detection	Species	Reference
Oxidative pentose phosphate pathway	Isotope dilution studies	Pea, soybean, yeast	^83^
Glycolysis	Cell biology, affinity purification mass spectrometry, isotope dilution studies	*Arabidopis thaliana*, human cell lines, human heart, potato, yeast, *Synecocystis* sp.	^57,84–86^
TCA cycle	Cell biology, affinity purification mass spectrometry, isotope dilution studies, site-directed mutagenesis, kinetic modelling, protein crystallisation	Arabidopsis, *Bacillus subtilis*, potato, *S. cerevisiae*, *E. coli*, rat	^40–42,45,67,82,87–91^
CoA channelling through FA synthesis	Substrate pool size quantification and enzyme kinetics	Pea and spinach	^92^
FA β-oxidation cycle	Protein crystallisation	*E. coli*	^93^
Polyketide pathway	Enzyme kinetic analysis	*Saccharopolyspora erythraea*	^94^
Purinosome	Cell biology, proteomics, mutant analysis, comparison of enzyme assembly with the rate of flux through the pathway	Human cell lines	^49–51,95^
Mitochondrial ETC complexes	Enzyme assays in the presence/absence of electron donors and inhibitors and genetically modified supercomplexes	Mouse fibroblasts	^96^
Photosynthesis			
(i) Light-harvesting complex	Spectroscopic analysis of isolated protein complexes and genetically modified complexes	*Chlamydomonas reinhardtii*	^97^
(ii) Calvin–Benson cycle	Isotope dilution, proteomics	*Synechocystis* sp.	^98^
Cyanogenic glucoside biosynthesis	Isotope dilution, enzyme kinetic analysis, co-purification studies	Sorghum	^99,100^
Phenylpropanoid biosynthesis	Channelling of labelled intermediates, cell fractionation experiments, co-immunolocalisation	Plants including Arabidopsis, buckwheat, petunia, maize and snapdraogon	^101,102^
Polyamine biosynthesis	Yeast two hybrid, co-immunoprecipitations, cell biology	Arabidopsis, *Leishmania donovani*	^103,104^
Branched chain amino acid metabolism	Protein association studies, site directed mutational analysis	Isolated proteins from human and rat	^105^
Alkaloid	Cell biology	*Catharanthus roseus*	^106^
Tetrapyrrole biosynthesis	Crystallisation and structure determination	*Synechocystis* sp.	^107^
Lignin	Genetic manipulation, mathematical modelling	*Medicago trucatula*	^68,108^
Cell wall degradation	Cell biology, proteomics, crystallisation studies	Arabidopsis, rice, anaerobic bacteria and fungi	^109^
Proline catabolism	Kinetic considerations, effects of mutating one of the enzymes on the other, modelling based on available crystal structures	*Thermus thermophilus*	^110^
RNA degradation	Cell biology, proteomics, crystallisation studies	*E. coli*, *Psuedomonas syringae*, *Rhodobacter capsulatus*, *Streptomyces coelicolor*, *Mycobacterium tuberculosis/bovis*	^111^

In this review, we consider the nature and functional consequences of organising enzymes into such assemblies, by drawing on recent experimental and theoretical advances. We will discuss two different types of assembly: (i) those built from specific pairwise interactions between sequential enzymes with complementary structural features; (ii) larger clusters formed from multiple copies of all enzymes of a pathway. These two types of assembly have different implications for substrate channelling, pathway flux and regulation of flux at metabolic network branch points and these will be considered in detail. We will illustrate each type of assembly by considering naturally occurring examples (focussing mainly on the TCA cycle and purine biosynthesis, where recent advances in the structure and function of the assemblies have been made) as well as examples from synthetic biology. Finally, we will consider the relevance of enzyme assemblies for metabolic engineering as well as discussing the crucial experimental evidence that will be required to fully understand the biochemical importance of this enigmatic phenomenon. Owing to space constraints, we cannot cover every reported dynamic superstructure nor can we discuss every feature of such assemblies but the interested reader is referred to several excellent recent reviews that cover these aspects in more detail^10–13^.

## The enigmatic and misunderstood phenomenon of substrate channelling

The most common explanation for the dynamic formation of enzymes into physical assemblies is that it promotes substrate channelling—that is, the facilitated transfer of the metabolite product from one enzyme to the next enzyme in the pathway without that metabolite equilibrating with the bulk aqueous solvent. This can be particularly beneficial if the channelled metabolite is labile or toxic, which is quite commonly the case in plant and fungal secondary metabolism^6^. It also has obvious implications for regulation of flux at branch points in the metabolic network—a channelled metabolite is not available for use by a competing enzyme at a branch point. This feature of substrate channelling will be discussed in more detail later.

But first we wish to address a major, and widely held, misconception about substrate channelling: which is that substrate channelling makes the metabolic pathway more efficient or kinetically superior^14^. Instinctively, this feels correct. Surely enzymes must work faster if substrates are passed between them without diffusing away into the bulk aqueous phase of the cell? However, for this to be true, diffusion would have to be a limiting factor in non-channelled reactions, and in most cases, it is not. Diffusion of metabolites is extremely fast in relation to the rate of catalysis by enzymes, even when the crowded and viscous environment of the cell is taken into account. Various calculations have been made that demonstrate this. For example, the calculated diffusion rate for collisions between enzymes and their metabolites is estimated to be 1000–10,000 times faster than the typical *K*_cat_/*K*_M_ value for enzymes of central metabolism^12^. Alternatively, it has been estimated the time taken for metabolites to diffuse between enzymes is one-to-three orders of magnitude faster than the average reaction turnover time for enzymes of central metabolism^12,15^. Similarly, a diffusion model was used to demonstrate that the concentration of product diffusing from an enzyme active site remains constant at distances of a few micrometres away^10^. Given that an enzyme concentration of 1 nM corresponds to an inter-enzyme distance of approximately 1 μm between enzymes, it is evident that diffusion is amply fast enough to evenly distribute metabolites between enzymes and does not limit their catalytic rate. In other words, it is reasonable to conclude that, diffusion is not the limiting factor for the rate of a reaction and hence metabolite channelling will not increase the rate of the reaction at steady state.

There are two exceptions in which metabolite channelling may accelerate the steady-state reaction rate. First, there are a small number of enzymes that are deemed to be catalytically perfect and whose *K*_cat_/*K*_M_ ratio is sufficiently high that the reaction does become limited by diffusion of the reactants^16^. A notable example in central metabolism is the glycolytic enzyme, triose phosphate isomerase. The second exception is if enzyme concentration is very low, meaning that the average distances between enzymes can increase to the point that the reaction is partially diffusion limited. This is considered to be a more relevant scenario for specialised metabolism of plants and fungi^12^.

## Why do synthetic enzyme assemblies go faster?

Over the past few years, there have been a growing number of experiments in which two or more enzymes of a metabolic pathway have been co-localised onto synthetic scaffold biomolecules^17,18^. These experiments are often predicated on the notion that enzyme proximity will lead to substrate channelling and that this will accelerate reaction rate. However, as we have already discussed, substrate channelling will not lead to a faster steady-state reaction rate than achieved by the freely diffusing enzymes unless the freely diffusing enzymes are at very low concentrations or are catalytically perfect enzymes. Given that these conditions do not apply to most of the published enzyme-scaffolding studies, then how to we reconcile the fact that almost all of these studies report several-fold enhancements of reaction rate for the scaffolded enzymes? Rate enhancements have been observed for scaffolds made from protein, lipid or DNA/RNA^19,20^ and for substrates with widely different physicochemical properties^21^.

**Fig. 1:**
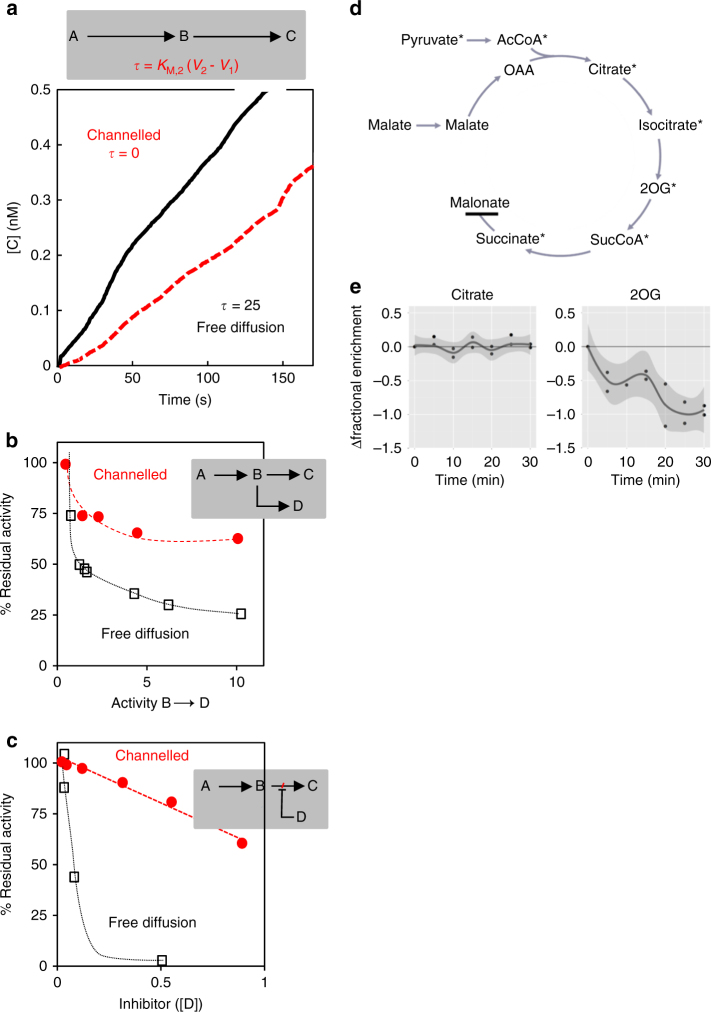
Methods of identifying substrate channelling. **a** Reaction scheme and depiction of transient time (*t*) analysis based on data from a channelled bifunctional thymidylate synthase-dihydrofolate reductase (TS-DHFR) and a freely diffusing monofunctional TS and DHFR (data from ref. ^64^). **b** Comparison of residual activity of a channelled or freely diffusing enzyme pair in the presence of a competing enzyme, for example, the malate dehydrogenase and citrate synthase couple in the presence or absence of aspartate aminotransferase, which competes for the metabolic intermediate oxaloacetate (data from ref. ^82^). **c** Comparison of residual activity of a channelled or freely diffusing enzyme pair in the presence of an inhibitor of the second enzyme, for example, the inhibition of the TS-DHFR cascade by the inhibition of DHFR by pyruvate (data from ref. ^64^). **d** Schematic representation of the isotope dilution experiment to assess the channelling of citrate and 2OG. ^13^C-labelled pyruvate was fed to isolated potato mitochondria and the label accumulation in succinate was monitored. The TCA cycle was inhibited by malonate to avoid the complication of multiple turns of the cycle. Non-labelled citrate and 2OG were added into the medium following the fractional enrichment in succinate reaching steady state. Asterisks show the expected fate of labelled carbon following the metabolism of pyruvate under the experimental conditions. **e** The result of isotope dilution experiments for citrate and 2OG. The time course plots show the fractional ^13^C enrichment in succinate following the addition of unlabelled citrate or 2OG at 0 min. The line is the smoothed conditional mean with the shadow representing a 95% confidence interval. The metabolite is considered not to be channelled when the confidence interval line falls below 0. Panels **a**–**c** has been adapted with permission from ref. ^10^. Panels **d**, **e** has been reproduced with permission from ref. ^41^

This apparent contradiction has prompted a reassessment of the nature of the rate enhancement in scaffolded reactions, using both modelling^22^ and experimental approaches^23,24^. This has provided a new perspective on the behaviour of co-localised enzymes that is relevant to our understanding of the function of in vivo enzyme assemblies. First, in enzyme-scaffolding experiments, reaction rate is typically monitored after initiating the reaction. Although the reaction is usually followed for tens of minutes thereafter, many reactions take hours to reach steady state in vitro. For example, a kinetic model of the commonly used glucose oxidase–horseradish peroxidase pair shows that, for experimentally relevant enzyme concentrations, the reaction takes >5 h to reach steady state^22^. In other words, much of the published data for scaffolded enzymes may be following the reaction during its ‘lag-phase’ prior to steady state. Therefore, the observed rate enhancement could be due to substrate channelling due to its effect of shortening the lag time (see Fig. 1a for a more detailed explanation). Nevertheless, there remain conceptual doubts as to whether channelling is likely in these systems^11,22^.

Moreover, it is also possible that alterations in enzyme kinetics could explain the enhancement of initial rate. Unfortunately, there is a lack of experimental assessment of this issue in the scaffolding literature. For a more detailed analysis of the effect of altered enzyme kinetics on reaction rates over time, we refer the reader to an excellent recent review^11^. In fact, there is evidence that immobilised enzymes can have superior kinetics^25^, although the underlying explanation for this remains unclear. It may be that immobilising the enzyme to a scaffold changes the local biophysical conditions resulting in a microenvironment that is closer to the intracellular environment. In other words, the enhanced kinetics of scaffolded enzymes are closer to in vivo kinetics than those measured in vitro for dilute solutions of purified enzymes. If true, this would have far-reaching consequences for kinetic modelling of metabolic networks. In a recent study of glucose oxidase and horseradish peroxidase enclosed in DNA ‘nanocages’, it was found that, when each enzyme was enclosed in a separate cage, the activity of the reaction was four-fold higher than for freely diffusing enzymes^26^. Importantly, the enclosed enzymes had a higher reaction than enzymes bound to a ‘half-cage’ that was open to the bulk solution, supporting a microenvironment-based explanation for altered enzyme kinetics. One intriguing suggestion as to the nature of this microenvironment effect is that the regularly spaced phosphates of the DNA scaffold decreases the local pH, which increase the reaction rate of this enzyme pair^23^. Indeed, tuning of the local pH environment around each enzyme in a reaction cascade has recently been shown to be an effective way of increasing reaction rate^27^. Another possible mechanism of altering of the kinetics is that enzyme assemblies may prevent compounds from exerting an inhibitory effect on the enzyme by restricting them from access to the active site of the enzyme^6^. It is thus likely that a range of biophysical/microenvironment effects could be at play to explain the increased initial reaction rate observed across multiple scaffold types and structures and different enzyme pairs.

## Substrate channelling mechanism

### Insights from the TCA cycle

Substrate channelling has benefits other than increasing reaction flux and there is abundant evidence that it does operate in naturally occurring enzyme assemblies. Perhaps the best-studied example of specific physical interactions between sequential enzymes associated with substrate channelling is the TCA cycle. Collectively, the body of work on the TCA cycle metabolon by Srere, Sumegi and their co-workers has been absolutely fundamental in setting our contemporary views concerning enzyme interactions and substrate channelling^2,28^. The general advantages of such channels have been mentioned above, namely, regulating metabolic flux at branch points as well as in specific cases being involved in the sequestration of labile and/or toxic intermediates. In addition, enzyme assemblies have been documented to influence allosteric effects (see, for example, ref. ^6^). There is a wealth of evidence from biochemical experiments for interactions between TCA cycle enzymes^2,28^ and between TCA cycle enzymes and inner mitochondrial membrane proteins^29–32^ or members of the mitochondrial carrier family^33,34^. In conjunction, there is a convincing body of evidence from enzyme kinetic and isotope labelling studies that strongly suggests metabolites are channelled between the enzymes of the cycle^31,35–39^. These classical studies have been bolstered by more recent work using modern techniques in cell biology and proteomics. For instance, binary interactions have been systematically assessed between *Bacillus subtilis* TCA cycle enzymes using a combination of bacterial two-hybrid analysis and affinity purification–mass spectrometry. Interactions were identified between six consecutive enzymes of the TCA cycle (linking fumarate to succinyl CoA), as well as interactions of these enzymes with phospho*enol*pyruvate carboxykinase and glutamate synthase^40^. In plants, a comprehensive range of techniques including affinity purification–mass spectrometry, split-luciferase and yeast two-hybrid assays have recently been utilised to demonstrate protein–protein interactions in the TCA cycle^41^.

Until recently, however, the mechanistic basis by which these enzyme–enzyme interactions facilitates substrate channelling has not been clear. Substrate channelling requires the metabolite product of one enzyme to be passed as substrate to the next enzyme in the pathway while being prevented from diffusing into the bulk solvent. In stable multi-enzyme complexes, the reaction intermediates are entirely enclosed in internal tunnels or chambers within the protein complex, allowing directed diffusion between active sites or the use of swing arms to physically mobilise intermediates or cofactors^10,15^. However, there is no evidence of such structures forming due to dynamic pairwise interactions of TCA cycle enzymes.

**Fig. 2:**
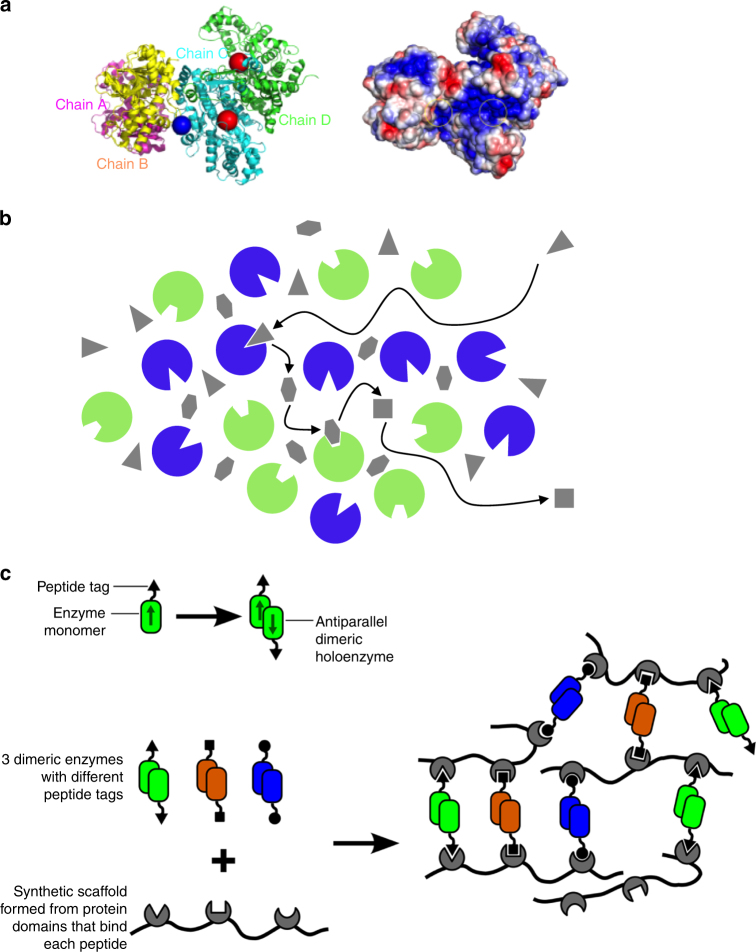
Mechanisms of substrate channelling in dynamic enzyme assemblies. **a** Direct channelling by electrostatic retention of the channelled metabolite on the surface of the enzyme complex. A structural model of the bovine malate dehydrogenase (MDH)–citrate synthase (CS) complex is shown. On the left, the polypeptides are illustrated as ribbon diagrams, with the MDH dimer shown in magenta and yellow and the CS dimer show in green and cyan. The blue circle shows where OAA molecules were initially placed in a Brownian dynamics simulation. Red circles show the active sites in the CS dimer. On the right, the surface structure of the complex is shown, with red and blue colours representing negative and positive electrostatic potential, respectively. Neutral regions are shown in white. Yellow circles indicate the positions of the adjacent MDH and CS active sites. **b** Probabilistic channelling within a large cluster of enzymes. Two enzymes are shown as green and blue circles. Metabolites are shown as grey polygons, with each shape representing a different metabolite. The arrows indicate the path taken by metabolites in a sequential conversion event by two enzymes. **c** Mechanism of enzyme cluster formation in synthetic scaffold-enzyme assemblies. When oligomeric enzymes are docked onto synthetic protein scaffolds via peptide tags, then interaction with more than one scaffold molecule are possible leading to the formation of a large aggregation of scaffolded enzyme complexes

A recent structural analysis of the malate dehydrogenase–citrate synthase–aconitase complex has provided insight into the potential mechanism by which substrate channelling could occur (Fig. 2a)^42^. In this study, in vivo crosslinking and mass spectrometry were used to establish a low-resolution structure of the complex. Crosslinking suggested that all eight enzymes of the cycle physically interact, but only the interactions between malate dehydrogenase and citrate synthase were sufficiently strong to allow their recovery in non-crosslinked samples. Using distance constraints derived from the crosslinking process, models of the malate dehydrogenase–citrate synthase–aconitase complex were proposed. The average distances between active sites were 35 Angstroms between malate dehydrogenase and citrate synthase and 50 Angstroms between aconitase and citrate synthase. Analysis of the modelled superstructure revealed that polar residues are considerably over-represented on its surface while less-polar residues tend to be even more buried than is usually the case. Moreover, these positively charge residues are arrayed within a groove that connects the malate dehydrogenase and citrate synthase active sites. Hence, complementary structural and charge features of the interacting enzymes provide a mechanism to promote efficient transfer of the negatively charged oxaloacetate intermediate between adjacent active sites. Electrostatic retention of the intermediate on the enzyme surface would not only reduce equilibration of the intermediate with the bulk, but theoretical models suggest that it would promote efficient transfer between the two enzymes^43^, provided that the electrostatic transfer groove is not too long^44^.

In a follow-up study, site-directed mutagenesis was performed on the highly conserved arginine residues located in the positively charged groove^42,45^. Mutation of one of these residues in the citrate synthase polypeptide to an uncharged reside (R65A) did not affect catalytic activity of the enzyme but reduced the probability of channelling from 0.99 to 0.023. Another intriguing feature which became apparent in these studies was that recombinant versions of malate dehydrogenase and citrate synthase self-assemble in vitro. From this work, it can be concluded that these enzymes have co-evolved complementary structural features that allow them to interact in such a way as to align surface features that may promote substrate channelling.

### Enzyme clusters or microdomains

One surprising feature of the more recent systematic studies of protein–protein interactions in the TCA cycle was the extensive interactions between non-sequential pathway enzymes in addition to the expected interactions between sequential enzymes^41^. It is conceivable that many of these interactions, which occur largely between regulatory subunits and catalytic subunits of different proteins, act as nucleation points that aid in the formation of the specific interactions between the sequential enzymes^41^. However, it is also possible that these additional interactions give rise to a different type of structure to the classical pairwise interaction: an enzyme cluster or enzyme microdomain. This is a larger assembly of multiple copies of each enzyme of a pathway as illustrated in Fig. 2b. This may or may not contain specific pairwise interactions within it and may or may not be ordered. The enzyme cluster leads to substrate channelling by a different mechanism and also has implications for reaction rate.

The concept of an enzyme cluster arose in the synthetic biology literature in which three enzymes of the mevalonic acid pathway were docked onto a protein scaffold by virtue of a peptide–protein interaction^46^. Recombinant enzymes were created with the relevant peptide tag at their C-terminus. However, because each of the holoenzymes is a homodimer with the two subunits in anti-parallel orientation, the scaffolded enzymes can interact with a second scaffold. Given the flexible nature of the scaffolds used, each enzyme does not necessarily interact with the same two scaffold molecules and it is therefore possible that a large cluster of physically connected enzyme-scaffold complexes can form^47^ (Fig. 2c).

In this cluster, substrate channelling can still occur but no longer requires specific pairwise interaction between sequential enzymes. Instead, the effect is probabilistic: when the metabolite product of an enzyme reaction diffuses away from the active site of one enzyme in a cluster, it has a much greater probability of encountering the next enzyme in the pathway than diffusing into the bulk aqueous solvent of the cell. This has been demonstrated in a reaction–diffusion model of a scaffolded enzyme system^48^. The model also suggested that the overall reaction rate would be enhanced. The explanation for the enhanced rate is simple: by bringing enzymes together into clusters, rather than having them distributed through the cell, the local concentration of enzymes is increased. This leads to a kinetic benefit for enzyme systems that are reaching substrate saturation. The model suggests that flux would increase by 6-fold for a 2-step pathway and over 100-fold for a 3-step pathway compared to freely diffusing enzymes within a cell^48^. Note that this is in contrast to the situation for enzyme assemblies formed solely from pairwise interactions where direct substrate channelling does not increase the steady-state reaction rate.

## The purinosome—a naturally occurring enzyme cluster?

**Fig. 3:**
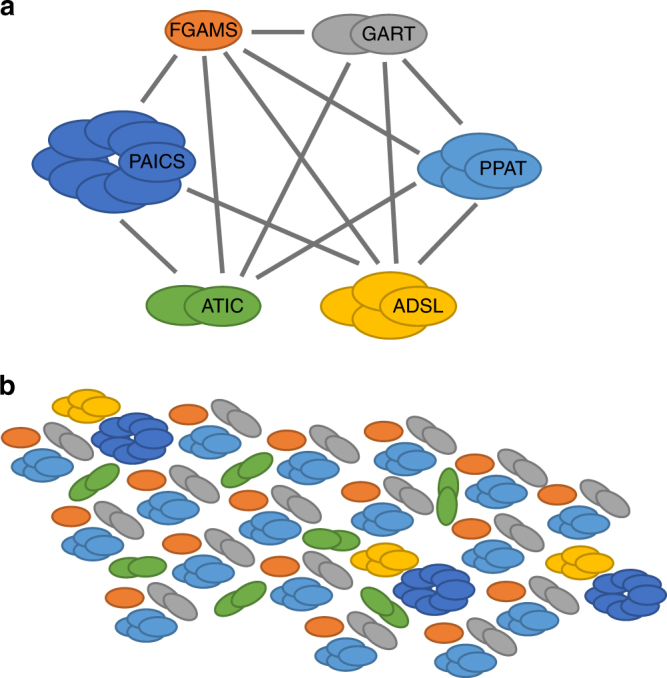
The purinosome—a naturally occurring enzyme cluster? **a** Protein–protein interactions within the purinosome. Each holoenzyme of the purine biosynthesis pathway is shown, with the number of subunits reflecting the dominant oligomeric state of each. The grey lines indicate experimentally demonstrated pairwise protein–protein interactions. PPAT phosphoribosylpyrophosphate amidotransferase, ADSL adenylosuccinate lyase, ATIC 5-aminoimidazole-4-carboxamide ribonucleotide formyltransferase/5′-inosinemonophosphate cyclohydrolase, FGAMS phosphoribosyl formylglycinamidine synthase, GART phosphoribosylglycinamide synthetase/phosphoribosylglycinamide formyltransferase/phosphoribosylaminoimidazole synthetase, PAICS phosphoribosyl aminoimidazole succinocarboxamide synthetase. **b** Potential organisation of the purine biosynthesis enzymes within the purinosome enzyme cluster. The first three enzymes of the pathway, PPAT, GART and FGAMS, form a core complex that interacts with an ADSL–PAICS pair and with ATIC. In this way, multiple copies of the core complex are assembled via interactions with ADSL–PAICS and ATIC

Recent work on purine nucleotide synthesis suggests that enzyme clusters are not only confined to synthetic enzyme assemblies but can also occur in the natural world. Purine nucleotides are a key component of DNA and RNA and also act as energy carriers and cofactors required to promote cell survival and proliferation. Remarkably, under cellular circumstances that lead to a high demand for purines, the enzymes of de novo purine biosynthesis cluster together into multi-enzyme complexes that have been dubbed purinosomes^49^. Confocal microscopy of HeLa cells revealed co-location of all six enzymes of the pathway in clusters of 0.2–0.9 μm in diameter under conditions where purine biosynthesis demand is high^50^. Proteomics has subsequently been used to define the nature of protein–protein interactions within the cluster demonstrating that a core complex which assembles in a step-wise fashion includes the first three enzymes in the pathway (Fig. 3a). Subsequent study of mutants of other enzymes in the pathway revealed a decreased purinosome formation suggesting that these proteins likely affect complex stability^51^. Further analysis of the assembly of the purinosome has implicated the Hsp70/Hsp90 chaperone machinery^52^ as well as casein kinase^53^, microtubules^53^ and G-protein coupled receptors^54^ in the process. Schematic representations of the purinosome complex typically show only a single copy of each holoenzyme (Fig. 3a). However, the purinosome complex as observed in vivo has an average diameter of 0.56 μm, which is sufficient room to contain many copies of the individual enzymes. If we assume that the 6 holoenzymes of the purinosome are spheres with an average diameter of 10 nm and are packed into a cubic volume at a packing density of 64% (the maximum packing density for irregular packing of spheres), then they would fit into a cube with edges of 17 nm in length, suggesting that 33 copies of each enzyme could be present (see Supplementary Note for details of this calculation). This is a minimum estimate given that the structure of the protein complexes is likely to allow closer packing density and protein stoichiometry is not necessarily equal, i.e. it is conceivable that many more copies of the three core-complex proteins could be present. Another way of looking at this is to consider that cells typically contain around 1 million protein molecules per μm^3^ ^55^. Hence the volume occupied by the purinosome (0.0092 μm^3^ for a sphere of diameter 0.56 μm) has room for 9200 individual proteins. Taking these calculations into account and what is known about the protein–protein interactions between the purine biosynthesis enzymes (Fig. 3a), we suggest that hundreds of copies of the three core-complex enzymes are present in the purinosome, brought together by interactions with the remaining enzymes of the pathway (Fig. 3b).

Further work is required to establish the exact architecture and size of the purinosome complex but it seems likely that the purinosome is a naturally occurring example of an enzyme cluster in which reaction rate is enhanced by increased enzyme concentration and substrate channelling is probabilistic rather than direct. Consistent with this, the formation of the purinosome in vivo is coincident with a 50% increase in measured flux through de novo purine biosynthesis^56^. The fact that the purinosome is formed when purine synthesis demand is high and is associated with increased flux suggests that purinosome formation is likely to be a means of controlling pathway flux.

## Dynamic enzyme assemblies control flux at network branch points

Whether one is considering enzyme assemblies formed by pairwise interactions or larger clusters of enzymes, an important feature of these complexes is that they effectively change the structure of the metabolic network due to substrate channelling. Specifically, they bypass branch points in the metabolic network: the channelled intermediate is prevented from being utilised in competing branch-point reactions.

Hence, the dynamic formation of enzyme assemblies is potentially an important regulatory feature allowing the relative flux in different branches of metabolic pathways to be controlled. This is critical when there is a necessity to rapidly increase the flux through one of the branches at the expense of the other. For example, substrate channelling between enzymes of glycolysis when located on the surface of mitochondria has been proposed to be a regulatory mechanism to ensure that sufficient pyruvate is supplied to the mitochondria when respiratory demand is increased, rather than allowing intermediates of glycolysis to be used for competing pathways, such as amino acid biosynthesis^57^.

Recently, the concept of substrate channelling as a branch-point regulatory mechanism has been directly experimentally tested by examining the effect of clustering of enzymes at a branch point between pyrimidine and arginine synthesis^48^. The branch point occurs at the metabolite, carbamoyl phosphate (CMP). Substrate channelling of CMP between the pyrimidine pathway enzymes CMP synthase (CMPS) and aspartate–CMP–transferase (ACMPT) would direct flux towards pyrimidine synthesis and prevent the arginine pathway enzyme ornithine–CMP–transferase competing for CMP. This flux change can be experimentally detected in bacteria as arginine pseudo-auxotrophy (i.e. growth is stimulated by the addition of arginine to the medium) and an insensitivity to the addition of uracil, a pyrimidine precursor. To test this, a genetic fusion of CMPS and ACMPT was expressed in a CMPS/ACMPT knockout strain of *Escherichia coli*. This did not result in arginine pseudo-auxotrophy, suggesting that the two enzymes lack structural compatibility/surface features to create channelling directly. However, when the fusion protein was more strongly overexpressed, protein clusters were visible under phase-contrast microscopy, assumed to be μm-scale assemblies of the CMPS–ACMPT fusion. In this situation, arginine pseudo-auxotrophy was observed, with the degree of growth stimulation by exogenous arginine correlated with the extent of protein cluster formation. This mechanism of flux control at branch points is now beginning to be incorporated into metabolic engineering experiments. For example, it was shown that flux from pyruvate to various fermentation end products in *E. coli* could be controlled by creating complexes of pyruvate kinase and the pyruvate-consuming enzymes of different fermentation branches^58^.

In the context of the purinosome and TCA cycle, there are known branch points in the local metabolic network that potentially could be controlled by this mechanism. In purine biosynthesis, the intermediate S-aminoimidazole ribonucleotide is a substrate for a reaction in the pathway of thiamine biosynthesis^59^. And the TCA cycle intermediate, oxaloacetate, is also used for aspartate biosynthesis^60^. Hence, in scenarios such as purine depletion where it is necessary to rapidly increase the flux of purine biosynthesis, the formation of the purinosome achieves this by two mechanisms: by increasing local enzyme concentration and by preventing competition for intermediates by branch-point pathways.

## Characterising the functional properties of dynamic enzyme assemblies

Enzyme assemblies have been characterised at the functional level, focussing mainly on substrate channelling, and at the structural level. It is clear that, if we are to better understand how these enzyme assemblies work, then the two facets need to be brought together. In the case of the TCA cycle, structural studies are bringing new insights into the potential mechanism of substrate channelling, but as of yet, the effect of mutation of structural features on substrate channelling have not been directly tested and so proof of the proposed mechanism is missing. In the case of the purinosome, there have been no direct tests of the effects of the complex on enzyme kinetics or substrate channelling. And more work is required to define the exact structure of the assembly and in particular whether it contains multiple copies of enzymes as we have suggested here. Another highly interesting aspect of the formation of dynamic enzyme assemblies that is receiving increasing attention is the influence of ionic and organic solute environments on their formation^45,61^. Intriguingly, high concentrations of compatible solutes not only promote enzyme folding and stability but also potentially alter the effective enzyme concentration that may affect oligomerisation and kinetics^62,63^, which may extend to associations between enzymes. The recent demonstration of a substrate driven chemostatic assembly of the enzyme cascade linking the first four activities of glycolysis (ref. ^61^), as well as the malate dehydrogenase–citrate synthase–aconitase complex assembly of the TCA cycle^45^, highlight the influence of small molecule metabolites on setting gradients aiding in dynamic enzyme assemblies. Although in a well-mixed cellular environment, it is unclear how relevant this gradient-driven assembly will be.

Going beyond the structural–functional level, the most important unanswered question of all is the role of enzyme assembly in the context of cellular biochemistry. We have proposed control of flux at metabolic branch points as a key role, but other advantages of such enzyme assemblies such as protection of labile reactants are also conceivable. Indeed, the latter has been suggested in the context of the purinosome where the product of the first enzyme of the pathway has a short solution half-life^49^. None of the proposed functional benefits of enzyme assemblies have been directly tested. To do this, one would ideally exploit the molecular mechanism that controls assembly and disassembly of the complex. Manipulation/mutation of this mechanism would allow the consequences of prevention of the enzyme assembly formation to be investigated and the biochemical role of the assembly to be established.

The other key question that needs to be addressed is how prevalent such enzyme assemblies are in nature. Protein–protein interaction studies have suggested that they are remarkably widespread and perhaps even the rule rather than the exception. But few of these interactions have been functionally assessed and this is largely due to the technical limitations and difficulties associated with classical methods of assessing substrate channelling. The methods available to biochemists for assessing substrate channelling are summarised in Fig. 1. For example, features of reaction kinetics such as the lag time (also known as the transient time, *τ*) can be used as an indirect method of demonstrating substrate channelling. Following initiation of a two-step reaction, there is a lag before the reaction reaches a steady state in which the intermediate metabolite concentration reaches a constant value dictated by the kinetics of the producing and consuming enzyme and the size of the reaction container. If channelling occurs, the reaction container volume is effectively reduced and the reaction therefore reaches steady state much more rapidly. In a perfectly channelled system, the lag time approaches zero (Fig. 1a). However, as already discussed, changes in kinetic properties and concentrations of the enzymes also affect the lag time and in many cases appropriate controls to account for this are missing^11^. An alternative kinetic method is to perturb the reaction from the bulk environment, as exemplified in Fig. 1b, c, which show the perturbation by a competing enzyme or the presence of an inhibitory molecule within the bulk environment, respectively. The first approach has been used to assess channelling mediated by a malate dehydrogenase–citrate synthase couple in the presence or absence of aspartate amino transferase^39^, while the second was applied to the bifunctional thymidylate synthase-dihydrofolate reductase in the presence and absence of trimethoprim and pyrimethamine^64^. Ultimately, these methods are generally limited to in vitro studies and are complicated by the fact that one is looking for the absence of an effect as evidence of substrate channelling^39^.

A more useful approach involves isotopically labelled substrates. In the ‘isotope dilution’ method, an isotope-labelled precursor to the pathway of interest is supplied to cells or tissues and the dilution of label in downstream pathway products following addition of an unlabelled pathway intermediate to the bulk solvent is monitored. Substrate channelling reduces or prevents equilibration of the bulk-solvent unlabelled intermediate with the equivalent channelled intermediate and so reduces or prevents isotope dilution^65^. The approach has two main limitations: first, many metabolic intermediates are not effectively taken up from the exogenous medium. This can be dealt with by permeabilisation treatments or expression of broad specificity transporters, as performed in studies of channelling in bacterial glycolysis^66^. The second problem is that the labelling patterns following addition of the unlabelled intermediate can be difficult to interpret, depending on the extent to which the enzymes under consideration are saturated with substrate, for example. Labelling patterns can be further confounded by cyclical pathways where multiple rounds of the cycle can lead to loss of label or excessive randomisation. The latter issue was addressed in a recent study of substrate channelling in the plant TCA cycle by using inhibitors to linearise the cycle. Isolated potato tuber mitochondria were supplied with ^13^C-labelled pyruvate or glutamate until accumulation of ^13^C in TCA cycle intermediates reached isotopic steady state (Fig. 1d, e). Then unlabelled intermediates of the TCA cycle were added and the dilution effect on labelling was monitored over time. For the pyruvate feeding experiments, the TCA cycle was linearised by inhibiting succinate dehydrogenase with malonate and, for the glutamate experiments, by inhibiting aconitase with fluoroacetate^41^. These experiments revealed dilution in 2-oxoglutarate, succinate and malate but none in fumarate or citrate, indicating channelling of these two intermediates between the respective enzyme pairs.

It should be obvious from these discussions that new methods are needed that would allow substrate channelling to be assessed in vivo, at a wider scale and with greater throughput. A new approach that has potential to achieve this has recently been described and is based upon the well-established methodologies of ^13^C-metabolic flux analysis (MFA). In MFA, a ^13^C-labelled metabolic precursor that is readily taken up is supplied and the pattern of redistributed label at isotopic steady state is analysed. Labelling patterns are then simulated in a metabolic network model and fluxes in the model iteratively varied until a good fit between the predicted and measured labelling patterns is achieved. Because substrate channelling changes the structure of the metabolic network where it bypasses branch points, the approach can be used to determine whether substrate channelling is required to account for the labelling patterns and, if so, can provide a quantitative estimate of the substrate channelled flux in vivo. This method was applied to heterotrophic Arabidopsis cell cultures and revealed substrate channelling of fumarate to malate but no channelled flux from 2OG or succinate to citrate^67^ in the plant TCA cycle.

A related modelling-based approach for quantitative assessment of channelling at the metabolic network scale is to construct an enzyme kinetic model and to assess whether the introduction of specific channelled pathways improves the ability of the model to predict observed data sets. This approach was used to assess channelling between two branch points in lignin biosynthesis in plants^68^. Depending on the operation of the channelled pathways, 19 different configurations of the lignin biosynthesis network could be formulated. These were each tested in a large set of Generalised Mass Action models that accounted for saturatable enzyme kinetics and aspects of regulatory crosstalk between the two channelled branches. The models were benchmarked against a data set of lignin content and monomer composition (guaiacyl (G), and syringyl (S) monomers) in transgenic alfalfa lines with reduced expression of different enzymes in the lignin pathway. The modelling suggested that channelling is necessary but not sufficient to explain the results of the transgenic plants and that specific crosstalk between the two channelled pathways is also required. Additionally, the modelling revealed that operation of the G-lignin channel was more important for an accurate description of the data than the S-lignin channel.

## Summary and outlook

The study of dynamically formed enzyme assemblies has a long history but it is only in recent years that structural features of these assemblies have begun to be elucidated. Coupled with a growing body of experimental work exploring the properties of synthetic enzyme assemblies, we are now able to provide a more integrated picture of the function of these transient enzyme complexes. Using the purinosome and the TCA cycle as examples, we have described two types of enzyme assembly, one formed from large clusters of multiple copies of enzymes and the other formed from single complexes of paired enzymes with complementary surface features that serve to direct diffusion of the channelled intermediate. We have argued that the role of these enzyme assemblies is regulatory. In the case of large enzyme clusters, the increase in local enzyme concentration is expected to increase pathway flux. And in both types of complex, effective channelling of intermediates (either directly between paired enzymes or probabilistically in large enzyme clusters) bypasses branch points in the local network, allowing flux through the channelled branch to be increased at the expense of the competing branch.

A number of other pertinent questions also remain to be addressed including the precise roles of sub-metabolons and the role of scaffolding proteins, described to be present in both the purinosome and TCA cycle assemblies, remains unclear. As does the role of membranes that are associated with both of these assemblies and many of the others listed in Table 1. Moreover, studies to define the influence of post-translational modifications such as reversible changes in structure or phosphorylation will likely be highly informative. Once the residues important for interaction and those important for protein modifications have been defined, the advent of genome-editing techniques should render the testing of their functional significance relatively facile.

A more complete understanding of the role of enzyme assemblies will also be crucial for engineering of metabolic networks and for the successful introduction of synthetic metabolic pathways into host organisms. Indeed it is possible that proteins within synthetic scaffolds are turned over at a decreased rate than the native enzymes. Failure to account for the presence of enzyme assemblies and substrate channelling will lead to flawed designs for engineered pathways and problematic interactions between synthetic pathways and host metabolism. For example, it has been suggested that substrate channelling in glycolysis prevents bacteria such as *E. coli* from utilising C5 sugars as fermentation substrates^12^, a major target for the biofuel industry allowing the use of plant lignocellulosic feedstocks. The argument is that the degradation products of C5 sugars are prevented from entering metabolism due to the channelling of glycolysis. Hence, it may be necessary to disrupt this endogenous channel in order to engineer bacteria that can efficiently ferment C5 sugars.

A final consideration is the potential link between substrate channelling and regulation of metabolism at the gene-expression level via metabolite signalling. Metabolite signalling is the greatest challenge for the metabolic engineer: current mathematical models of the combinatorial and non-linear gene-regulatory responses to changes in metabolite concentration are insufficient to allow accurate predictions of the outcome of genetic intervention in the metabolic network^69^. A recent breakthrough study of transcriptional regulation of metabolism in *E. coli* revealed that most of the metabolite feedback to the genetic regulatory system occurred via just three metabolites—cyclic AMP, fructose 1-phosphate and fructose 1,6-bisophosphate^70^. Given that substrate channelling effectively renders channelled metabolites invisible to metabolite signalling systems, this remarkable discovery may point towards a greater extent of substrate channelling in central metabolism than has hitherto been suspected, with the channelling effectively directing metabolic fluxes between a small number of non-channelled metabolite nodes upon which regulation is focussed (Box 1).

### Box 1. An evolutionary perspective of enzyme complexes

Here we review literature concerning the evolutionary histories of dynamic assemblies of enzymes and stable multi-enzyme complexes. More research of an evolutionary bent has been carried out on the latter—many of which are highly conserved throughout evolution. For example, a pioneering study suggested that the α-ketoacid dehydrogenase complexes appeared early in evolution, being found in aerobic members of the archaea, bacteria and eukaryotes^71^. Another multi-enzyme complex that has been subject to particular scrutiny is the fatty acid synthase complex (FAS)—of which two strikingly different types have evolved in eukaryotes: the metazoan and fungal FAS^72^. The metazoan FAS is a 540-KDa homodimer that shares a common architecture with bacterial polyketide synthases^73^, while the fungal FAS forms a 2.6-MDa assembly comprising 48 functional domains and, alongside the homologous CMN-FAS recently described in *Corynebacterium*, *Mycobacterium* and *Nocardia*^74^, constitute some of the most complex biochemical protein machineries known^75^. A recent study identified bacterial non-canonical fatty acid synthases and *trans*-acting polyketide enoyl reductases as potential ancestors of the scaffolding regions with striking conservation of insertions to scaffolding elements despite minimal sequence identity^72^. In contrast, plants and many bacteria harbour only monofunctional FASs^76^. Similarly, the penta-functional Arom complex of shikimate biosynthesis appears likely to have evolved via gene fusion with the enzymes of most other species being monofunctional^9^. A final multi-enzyme complex that merits discussion is that of tryptophan synthase^77^. In their study, Leopoldseder et al. suggest that it is plausible to assume that sophisticated multi-enzyme complexes evolve via stepwise association of protein subunits (see Supplementary Figure 3)^78^.

The evolution of metabolons is much less studied. However, as indicated in Table 1, these display a similar range of conservation throughout evolution as the stable multi-enzyme complexes. In particular, metabolons within the TCA cycle and glycolysis are highly conserved. Others, such as the urea/polyamine metabolon, are found, at least in part, in several species. Other metabolons, especially those involved in plant and fungal specialised metabolism, are far more restricted in their species range. However, despite the widespread occurrence of metabolons (dynamic enzyme assemblies) and stable multi-enzyme complexes, there is no reason to suspect that the evolution of the former is an obligatory step towards the evolution of the latter. Indeed, it is likely that the two types of enzyme assemblies have evolved to play quite distinct functions from one another.

The presence of protein aggregates has been argued to be under negative selection. However, protein aggregation may also be unavoidable (for example, owing to the limited solvency capacity of the cell in relation to the protein concentration) and therefore functional organisation around such aggregates may represent an evolutionary compromise^79^. Given that many oligomeric proteins may adopt multiple structural conformations, there may be unique opportunities for mutation to lead to the formation of new protein–protein contact points. While in many cases new aggregations will be deleterious, some will be neutral and even ultimately beneficial to the cell^79^, in which case one would anticipate their stabilisation. Recent studies of protein interaction data in the context of the evolutionary properties of the interaction network have left several unanswered questions^80^. Most pertinent of these being (i) to what extent do protein interactions act as constraints during the evolution of the protein sequence; (ii) what role do transient or obligate interactions play in these constraints; (iii) are mutations in the binding site of an interacting protein correlated in those in the binding site of its partner. Intriguingly, a study aimed at addressing these questions observed that residues at the interfaces of obligate complexes evolved at a relatively slower rate allowing them to co-evolve with their interacting partners. In contrast, where the protein–protein interactions are transient, the rate of residue substitution is much higher^81^. These observations are thus in line with our suggestion that multi-enzyme complexes and metabolons evolve separately and result in distinctive repurposing of the ancestral enzymes, from one another. We propose that the key functional discriminator is the requirement for access of competing enzymes to the channelled metabolite so that metabolic branch points can operate. This would require disassembly of the enzyme complex that supports substrate channelling and would provide a selective pressure against the evolution of a stable multi-enzyme complex for that set of reactions. In other words, stable muti-enzyme complexes can only evolve where there are no other cellular reactions that requires access to the channelled metabolite.

## Electronic supplementary material

Supplementary Information
