# Supplementary material for: The role of dynamic enzyme assemblies and substrate channelling in metabolic regulation
Source: Nat Commun. 2018 May 30;9:2136. doi: 10.1038/s41467-018-04543-8 (PMC5976638; doi:10.1038/s41467-018-04543-8)
Supplement: Supplementary file 1 — Supplementary Information [file 41467_2018_4543_MOESM1_ESM.pdf]

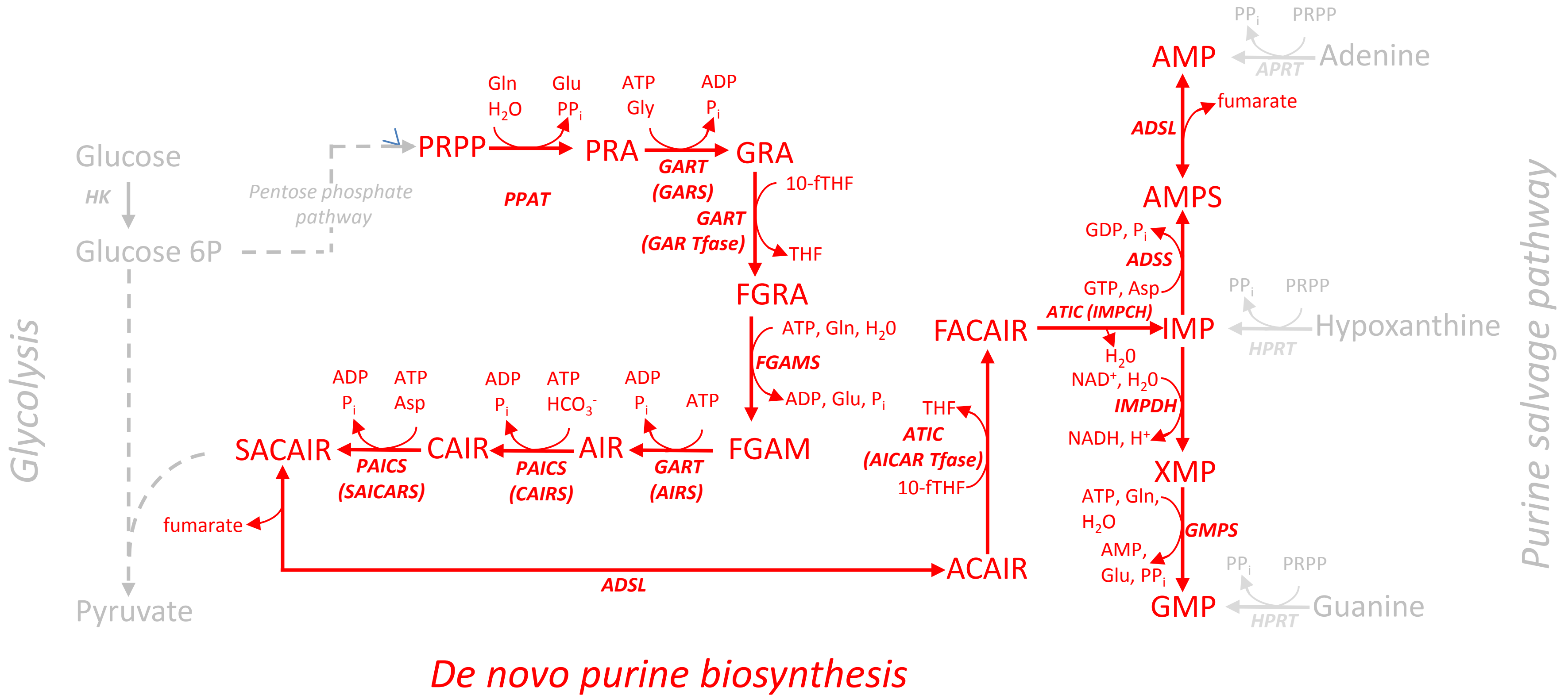

Supplementary Figure 1. Purine metabolism. The *de novo* pathway in humans consists of ten reactions (red) that transform phosphoribosylpyrophosphate (PRPP), generated via the pentose phosphate pathway (blue) into inosine 5`-monophosphate (IMP). Six enzymes catalyze these steps PRPP amidotransferase (PPAZ), trifunctional[phosphoribosylglycinamide synthase (GARS) /phosphoribosylglycinamide formyltransferase (GAR Tfase)/ phosphoribosyl formylglycinamodine synthetase (AIRS)], bifunctional [phosphoribosyl aminoimidazole carboxylase (CAIRS)/ phosphoribosyl aminoimadizole succinocarboxamide synthase (SAICARS)] PAICS, adenosuccinate lyase (ADSL) and bifunctional [5-aminoimadizole-4-caboxamide Ribonucleotide formyltransferase (AICAR Tfase)/ IMP cyclohydrolase (IMPCH)] ATIC. The pathway cofactor 10-formyltetrahydrofolate (10-fTHF) is a product of C1 metabolism. SAICAR activates glyolysis via allosteric effects on pyruvate kinase, AICAR is also a byproduct of histidine biosynthesis. Purine biosynthesis downstream of IMP requires the activities of IMP dehydrogenase (IMPDH), and GMP synthase (GMPS) to make GMP or adenylosuccinate synthase (ADSS) and ADSL to generate AMP. Purine salvage can also be used to generate IMP and GMP using hypoxanthine-guanine phosphoribosyl transferase (HPRT) and AMP via adenine phosphoribosyl transferase (APRT).

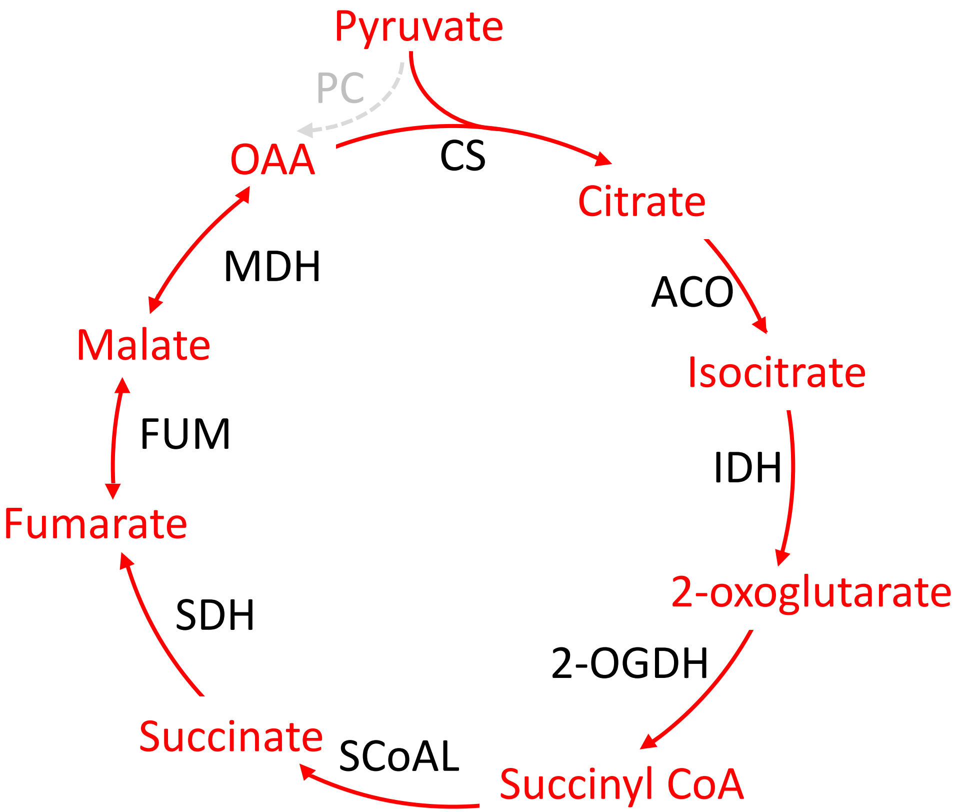

Supplementary Figure 2. The tricarboxylic acid (TCA) cycle. The eight core reactions of the TCA cycle which are common to nearly all organisms. Abbreviations: citrate synthase (CS); aconitase (ACO); isocitrate dehydrogenase (IDH); 2-oxoglutarate dehydrogenase (2-OGDH); Succinyl CoA ligase (SCoAL); succinate dehydrogenase (SDH); fumarase (FUM); malate dehydrogenase (MDH); pyruvate carboxylase specific to glial cells; OAA, oxaloacetate.

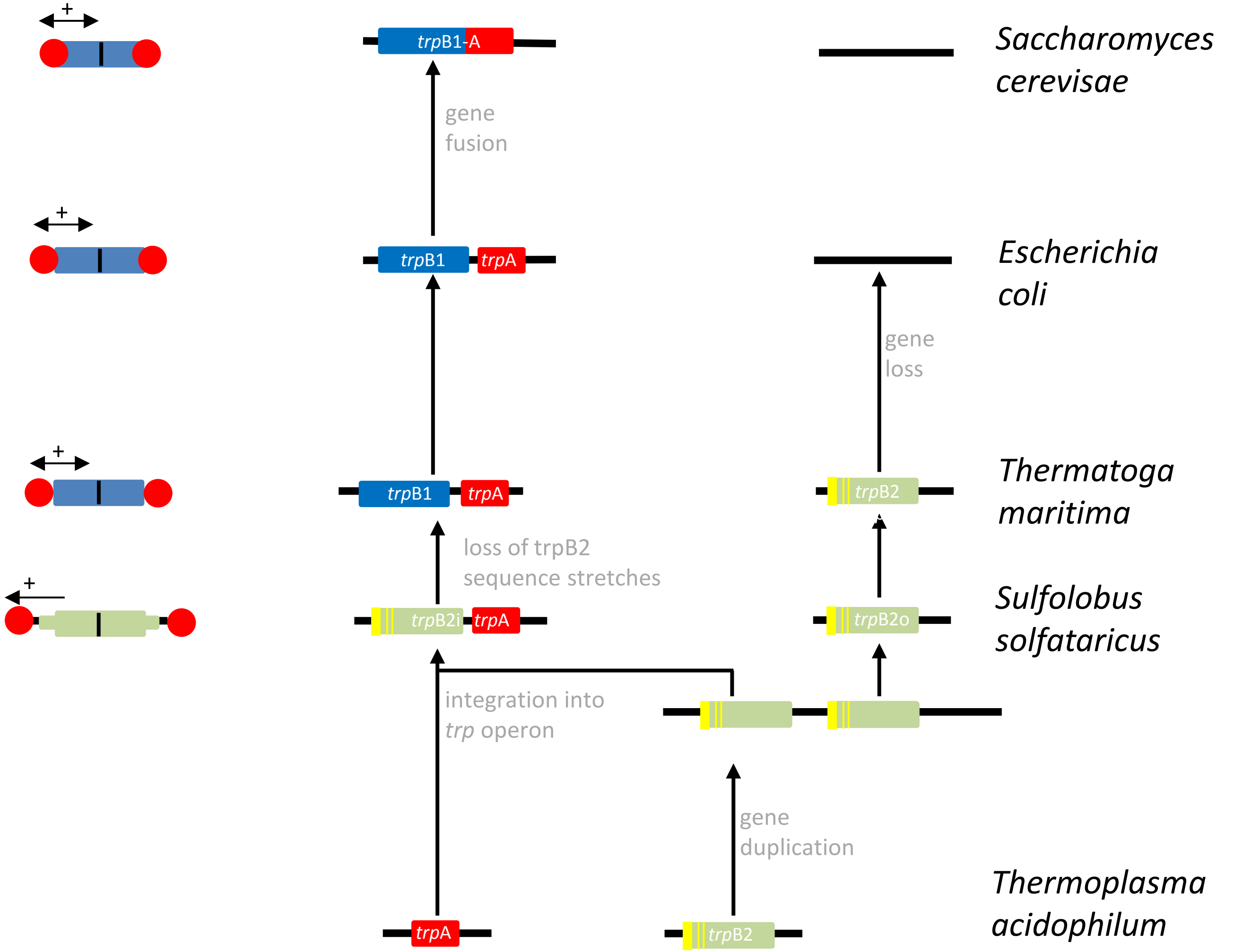

Supplementary Figure 3. Schematic of the evolution of the tryptophan synthase complex. The organisms listed provide examples of the given trpA and trpB Gene organizations and the interactions between TrpA and TrpB subunits. Figure adapted with permission from reference [107]. Copyright 2006 American Chemical Society.

Supplementary Note Calculation of potential protein copy number within the purinosome.

The purinosome consists of 6 holoenzymes with oligomeric states that include one monomer, two dimers, two tetramers and one octomer [1]. The average oligomeric protein has a diameter of 10 nm (http://book.bionumbers.org/how-big-is-the-average-protein/).

The question is how many protein oligomers can fit inside a body the size of a purinosome which has been reported to have an average diameter of 0.56 μm [1].

One can consider this as sphere packing problem. Given that the exact structural architecture of the purinosome is not known, let us assume that the proteins spheres are irregularly packed into a cube of length 0.56 μm. The maximum packing density for irregularly packed spheres is 64 % [2].

The packing density (P) is given by:

$$P = \frac{V_{sphere}}{V_{cube}}$$

Where V_{sphere} is the total volume of spheres in a cube and V_{cube} is the volume of the cube.

From this we can calculate the size of cube that the 6 holoenzymes of the purinosome can pack into:

$$V_{cube} = \frac{V_{sphere}}{P}$$

$$V_{sphere} = \frac{4}{3}\pi * 5^3 * 6 = 3141.6\, nm^3$$

$$\therefore V_{cube} = \frac{3141.6}{0.64} = 4908.8\, nm^3$$

Length of each side of cube = $\sqrt[3]{4908.8} = 17.0\, nm$

Therefore, in a cube of length 0.56 μm (the purinosome), we can fit 33 (0.56/0.017) cubes containing a single copy of each enzyme. In other words, 33 copies of each enzyme can be fitted within the purinosome body, assuming irregular packing of the enzymes.

Supplementary References

[1] Pedley, A. M. & Benkovic, S. J. A New View into the Regulation of Purine Metabolism: The Purinosome. *Trends in Biochemical Sciences* **42**, 141-154, doi:10.1016/j.tibs.2016.09.009 (2017).

[2] Song, C., Wang, P. & Makse, H. A. A phase diagram for jammed matter. Nature **453:** 629–632. (2008) doi:10.1038/nature06981
